# Retrospective study of spirocercosis in coyotes (*Canis latrans*) in Costa Rica: epidemiology, pathological findings, and molecular characterization of *Spirocerca lupi*

**DOI:** 10.1186/s13071-025-07030-4

**Published:** 2025-10-27

**Authors:** L. Mario Romero-Vega, Alicia Rojas, Mario Santoro, Flavia Occhibove, Joby Robleto-Quesada, Gabriela Benavides, Isabel Hagnauer, Andrés Moreira-Soto, Alejandro Alfaro-Alarcon

**Affiliations:** 1 Department of Pathology, School of Veterinary Medicine, National University, Heredia, Costa Rica; 2 Institute of Pathology, University of Veterinary Medicine Hannover Foundation, Hannover, Germany; 3 Laboratory of Helminthology, Faculty of Microbiology, University of Costa Rica, San José, Costa Rica; 4 Center for Research in Tropical Diseases (CIET), Faculty of Microbiology, University of Costa Rica, San José, Costa Rica; 5 Department of Integrative Marine Ecology, Stazione Zoologica Anton Dohrn, Napoli NA, Italy; 6 Clinic Laboratory, Guapiles Hospital, Caja Costarricense de Seguro Social, San José, Costa Rica; 7 Rescate Wildlife Rescue Center, Fundacion Restauracion de La Naturaleza, Alajuela, Costa Rica; 8 Berlin Institute of Health, Institute of Virology, Charité-Universitätsmedizin, Berlin, Germany

**Keywords:** Costa Rica, Coyote, Histopathology, *Spirocerca*, Pathology

## Abstract

**Background:**

*Spirocerca lupi* is a nematode that infects domestic dogs and wild carnivores.

**Methods:**

This study retrospectively analyzed postmortem records between 1989 and 2024 to assess *S. lupi*-associated lesions in coyote necropsies. In addition, it conducted molecular characterization of *18S* rRNA and cytochrome oxidase subunit 1 (*cox1*) gene fragments of larvae found at necropsies. Fecal samples from free-ranging coyotes were molecularly examined for *S. lupi* *18S* DNA.

**Results:**

Of the 39 coyote cases, 33 (84. 6%) presented *S. lupi*-associated lesions. A significant association was observed between the presence of *Spirocerca* nematodes and the development of esophageal granulomas and aortic aneurysms. In addition, an atypical case of spinal cord invasion was documented, representing the first reported occurrence of this condition in coyotes. Out of all fecal samples tested, 4.6% were positive for *S. lupi* infection.

**Conclusions:**

The obtained *cox1* sequence revealed a complete similarity to *S. lupi* isolated from the Andean fox *Lycalopex culpaeus* from Peru, suggesting its transmission between wild canid populations. These findings indicate that coyotes play a significant role in *S. lupi* transmission dynamics and highlight the need for further research on the ecological interactions between domestic and wild canids in Costa Rica.

**Graphical abstract:**

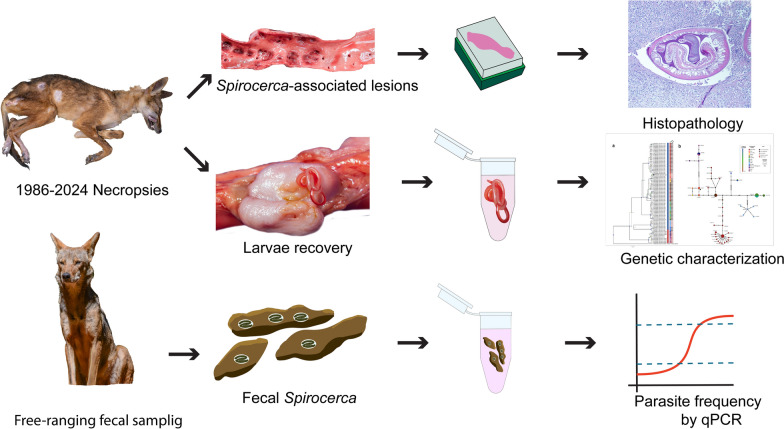

**Supplementary Information:**

The online version contains supplementary material available at 10.1186/s13071-025-07030-4.

## Background

Spirocercosis is a parasitic disease caused by spirurid nematodes of the *Spirocerca* genus (family Spirocercidae). The genus *Spirocerca* comprises three recognized species, *Spirocerca lupi*, *S. vigisiana*, and *S. vulpis* [1, 2]*. Spirocerca lupi* infects primarily domestic dogs and a wide range of wild canids as definitive hosts; in contrast, *S. vigisiana* and *S. vulpis* have been found exclusively in the red fox (*Vulpes vulpes*) [1]. There are two forms of transmission of *S. lupi* to the definitive host: (i) by ingestion of coprophagic beetles of the Scarabaeidae family [3], which serve as intermediate hosts, or (ii) by ingestion of paratenic hosts, such as lizards or shrews infected with parasite larvae [2]. Spirocercosis is known to cause distinctive pathological changes considered to be pathognomonic in its definitive hosts [4]. When the definitive host ingests the infected intermediate/paratenic host, the released larvae enter the gastric mucosa and migrate through the gastric arteries to the caudal thoracic aorta moulting two times, then reach the esophagus where the parasites mature and induce the formation of a nodule with a fistulous opening into the esophageal lumen from which the mature females lay their eggs [5]. The larval migration can lead to arteriosclerosis of the gastric arteries and aortic aneurysms and/or granulomatous inflammation, characterized by varying degrees of granulation tissue [2]. Esophageal penetration of the parasite induces a significant inflammatory reaction, primarily involving macrophages, neutrophils, and lymphocytes [2]. Chronic inflammation and prolonged exposure to the parasite can lead to the development of sarcomas, often fibrosarcoma or even osteosarcoma [6]. Spirocercosis is known to cause distinctive pathological changes considered to be pathognomonic in its definitive hosts [4].

Most reports of infection with *S. lupi* involve Neotropical carnivores, including the Andean fox (*Lycalopex culpaeus*) [7], the maned wolf (*Chrysocyon brachyurus*) [8], the bush dog (*Speothos venaticus*) [9], the raccoon (*Procyon lotor*) [10], the gray fox (*Urocyon cinereoargenteus*) [11], and the coyote (*Canis latrans*) [12]. Molecular characterization of *Spirocerca* spp. in these hosts is scarce, with most of the sequences available in GenBank derived from infections in domestic dogs [13]. Intralesional larval characterizations in wildlife are rarely performed, and, to our knowledge, the molecular characterization of *S. lupi* adults recovered from pathologic lesions in coyotes has not been conducted.

Coyotes, which have adapted well to anthropogenic pressures [14], have expanded their range from North America into Central and South America in the last 50 years [15]. In some countries, overpopulation has led to nonlethal control measures for this species [16]. Although there is currently no evidence of overpopulation in Costa Rica, urban sightings have recently become more frequent [17]. This increased urban presence could pose an infection risk to domestic dogs, as both species may frequent urban parks [18]. Understanding the health status of coyotes is crucial for predicting potential pathogen exchanges among coyotes, domestic dogs, and other wild canids [19–21].

This retrospective study analyzed postmortem data from coyotes submitted to the Pathology Laboratory at the School of Veterinary Medicine, National University of Costa Rica. It provided the first molecular characterization of intralesional *S. lupi* in wild coyotes. The fecal material obtained from a comprehensive population of wild coyotes was also investigated for *Spirocerca* infection.

## Methods

### Retrospective and histopathological analysis

Retrospective cases of free-ranging coyotes obtained in Costa Rica from 1989 to 2024, and submitted as biopsy or necropsy material to the Pathology Department, School of Veterinary Medicine, National University of Costa Rica (PD-EMV), were reviewed for a better appreciation of spirocercosis occurrence and associated lesions. Newly prepared hematoxylin and eosin-stained slides from the preserved paraffin blocks of selected tissues, including aorta, heart, lungs, kidneys, esophagus, stomach, and small and large intestine, were examined. A positive spirocercosis coyote was defined by the observation of an adult or immature individual of *Spirocerca* spp., their eggs, or pathognomonic lesions of past infection (i.e., esophageal nodule, esophageal sarcoma, aortic aneurysm) [22] in a single histologic slide of that retrospective case. For cases from 2018 to 2023, suspected *S. lupi* L3 and L4 larvae were collected in 70% ethanol for molecular characterization.

### Molecular identification of larvae

DNA was extracted from fragments of four larvae collected at necropsies of *Spirocerca*-associated lesions using a DNeasy Blood and Tissue Kit^©^ (Qiagen™, Hilden, Germany) according to the manufacturer’s instructions. Molecular identification of the specimens included the amplification of a region of the *18S* rRNA gene and an area of the mtDNA *cox1* gene. The *18S* rRNA region was amplified with two primer sets, amplifying partially overlapping regions: ﻿Nem18S-F1 (5′-CGCGAATRGCTCATTACAACAGC-3′) and Nem18S-R1 (5′-GGGCGGTATCTGATCGCC-3′) [23]; Nem18S-F2 (5′-CGAAAGTCAGAGGTTCGAAGG-3′) and Nem18S-R2 (5′-AACCTTGTTACGACTTTTGCCC-3′) [24]. The polymerase chain reaction (PCR), with a volume ﻿of 25 μL, included 0.6 μL of each primer (10 mM), 3 μL of MgCl2 (25 mM) (Promega™, USA), 5 μL of 5× buffer (Promega™), 0.6 μL of dNTPs (10 mM) (Promega™), 0.3 μL of Go-Taq Polymerase (5 U/μL) (Promega™), and 2 μL of total DNA template, adding ultrapure water to reach the final volume. Thermocycling conditions followed parameters published elsewhere [24]. The *18S* rRNA successful PCR products were purified using Agencourt AMPure XP^©^ (Beckman Coulter™, USA), following the standard manufacturer-recommended protocol. Partial *cox1* gene fragments were amplified using JB3 (5´-TTTTTTGGGCATCCTGAGGTTTAT-3´) and JB45 (5´-TAAAGAAAGAACATAATGAAAATG-3´) primers [25]. Final volume of PCR was 25 μL, which included primers at a final concentration of 400 nM, DreamTaq Master Mix^©^ (Thermo Scientific™), and 3 μL of DNA template with ultrapure water to reach the final volume. PCR conditions were those described previously [23] and later, PCR products were analyzed using 1.5% agarose gels. Clean PCR products were submitted for Sanger sequencing from both strands (Macrogen™ Europe, Holland). The obtained contiguous sequences were assembled and edited using Unipro UGENE™ [26]. Sequence identity was checked using BLASTn [27].

#### Phylogenetic analysis

Sequences were inspected and primers removed using MEGA7 [28]. Reference sequences of *S. lupi* and *S. vulpis* available in GenBank were aligned with the ones obtained from coyotes from Costa Rica using MEGA7 [28]. The best nucleotide substitution model for the *18S* and *cox1* was the Kimura-2 parameter with gamma distribution as predicted with JModelTest [29]. A Bayesian inference (BI) phylogenetic tree was built for each dataset using the BEAST version 2.5 package [30]. Accordingly, the *18S* and *cox1*.fas files were uploaded to BEAUTi version 2.7.7 with the respective nucleotide substitution model, 10 Monte Carlo Markov chains, trees were logged every 10 chains, and 10% was set as burning. Next, the BI trees were analyzed using BEAST version 2.7.7 and summarized with TreeAnotator version 2.7.7 after checking that all effective size population parameters were larger than 300 in Tracer version 1.7.2. Then, trees were visualized in FigTree version 1.4.4, and a Templeton Crandall Sing haplotype network was built for *cox1* data with PopArt30 using a 95% connection limit.

### Fecal sampling

Coyote scat samples were collected from the Guanacaste and Central Conservation Areas of Costa Rica during the rainy season (April to December) of 2021 on the basis of their distinctive macroscopic characteristics [31]. Sample collection permits in national parks were granted by SINAC-ACC-1771–2020 and the Biodiversity Commission from the University of Costa Rica, permit 265–2020. An aliquot of each sample was immediately frozen at −20 °C and stored until further molecular analyses. A total of 137 samples of coyotes were tested in this study using real-time PCR (Supplementary Table S1).

#### DNA extraction and coyote species confirmation

Approximately 0.2 g of each fecal sample was subjected to total DNA extraction using the QIAamp DNA Stool Mini kit^®^ (Qiagen^©^, Germany) according to the manufacturer’s instructions, with the following modifications: an additional step was included in which five glass beads were added after incubation at 95 °C to facilitate the breaking of helminth eggs. Once the beads were added, the tubes were vortexed for 10 min [32]. The samples were eluted twice in 100 µl of buffer AE for a final volume of 200 µl. After extraction, the DNA was stored at –20 °C until further experiments were carried out. To ensure collected samples were indeed from *Canis latrans*, we used a PCR–restriction fragment length polymorphism (RFLP)-based method described elsewhere [33].

#### Quantitative (q)PCR for detection of *Spirocerca lupi* eggs in feces

Real-time amplification for detection of *S. lupi* eggs and melt curve analysis were performed using the Applied Biosystems StepOne™ software (Applied Biosystems, USA) for the amplification of a 270-base pair (bp) fragment of the *18S* gene of *S. lupi* using Sl18S-F (5′-AAGCTCCGACTTTTGGACGA-F′) and Sl18S-R (5′- GTCACTACCTCCTCATGCCG-3′) primers [34]. The reaction was performed in a final volume of 20 μL containing 1 μl of each primer at 500 nM final concentration, 10 μl of SYBR Green Master Mix^©^ (Thermo Fisher Scientific Inc™.), 5 μl of ultrapure water (UPW), and 3 μl of DNA. The amplification reaction consisted of an initial denaturation at 95 °C for 4 min, followed by 50 cycles of 95 °C for 10 s, annealing at 59 °C for 15 s, and extension at 72 °C for 10 s. A high-resolution melt (HRM) curve was generated from 78 °C to 88 °C with 0.1 °C/s increments. Each sample was analyzed in triplicate. Adult *S. lupi* DNA was used as a positive control, and UPW as a non-template control (NTC) for all runs. Samples were considered positive if they exhibited a melting temperature (Tm) of 83.6 ± 0.2 (°C) in two or more replicates. To validate positive results from qPCR, amplicons were purified using ExoSAP^©^ purification enzymes (Applied Biosystems) and Sanger sequencing (Macrogen Inc., South Korea). Samples were considered positive for *S. lupi* when the obtained amplicon had at least 97% sequence identity to reference sequences in GenBank (accession no. MG957121).

### Association between spirocercosis and pathological and demographical variables

To assess the associations between *S. lupi* diagnostics and various pathological conditions, we analyzed a dataset containing categorical variables related to age, sex, granuloma presence, nematode infection, tumors, aortic aneurysms, and gastric artery conditions. Statistical analyses were performed using chi-squared tests to determine the significant associations (*P* < 0.05), and Cramér’s V test was used to quantify the strength of these associations. Data were processed and visualized using R (version 4.4.3) statistical software, generating correlation matrices and significance plots.

## Results

### Anatomopathological study

**Fig. 1:**
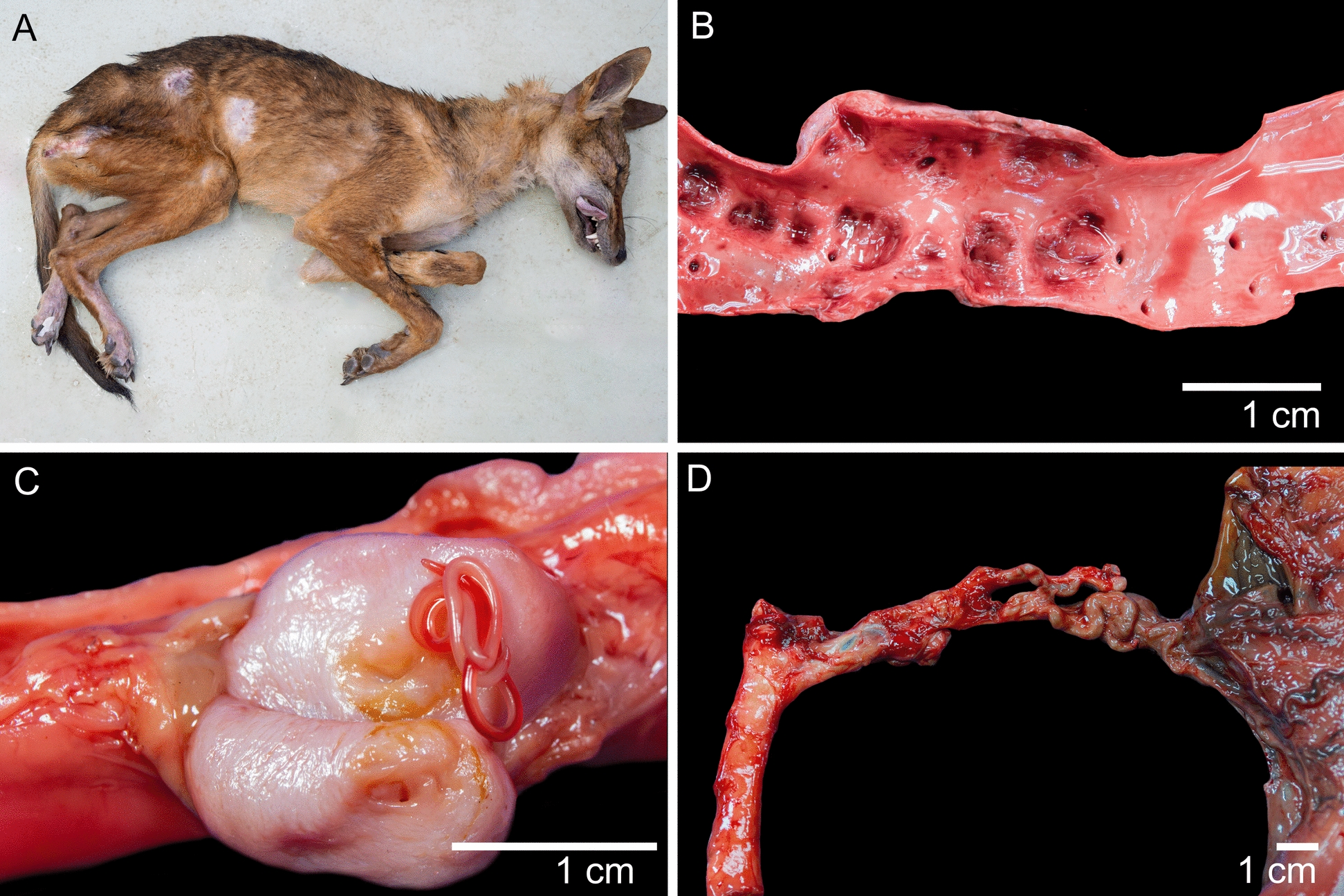
Principal *Spirocerca*-associated lesions. **A** Poor body condition in a positive animal. This particular animal was positive for intravertebral spirocercosis, which explains the cutaneous ulceration in the posterior limbs associated with proprioceptive deficit. **B** Aortic aneurysm. Severe weakening and dilatation of vascular wall. **C** Macroscopic view of aortic nodule containing sub-adult *Spirocerca* larvae. **D** Arteriosclerosis of gastric arteries

A total of 39 coyotes were submitted to the Pathology Department of the School of Veterinary Medicine (PD-EMV) from 1989 to 2024. Animals used for necropsies had poor body condition, based on the absence of subcutaneous fat [35] (Fig. 1A). A total of 33 coyotes had lesions associated with *S. lupi* infection, including aortic aneurysms (Fig. 1B) (84%), granulomas/nodules (66%) (Fig. 1C), or fibrosarcomas (< 1%), with aortic aneurysms being the most common lesion (Table 1). Except for one case of fibrosarcoma, no other type of neoplasia was diagnosed. Arteriosclerosis of gastric arteries was found only in one case, characterized by tortuous and rigid gastric arteries (Fig. 1D). No ruptured aneurysms nor fatal hemothorax were reported. *Spirocerca* larvae were found in the aorta or esophagus of 22 coyotes. Adult migration to the last thoracic vertebra was an incidental finding in one of the necropsied animals.

**Table 1:** *Spirocerca*-associated lesions in necropsied coyotes

Year	Age	SAL	Esophageal nodule	Intralesional larvae	Neoplasia	Aortic aneurysm	Gastric artery
2024	Adult	+		+		+	
	Adult	+				+	
2023	Adult	+				+	
	Juvenile						
2022	Juvenile	+				+	+
2021	Adult	+	+	+		+	+
	Adult	+	+	+		+	
2020	Adult	+	+	+		+	
	Juvenile	+	+	+		+	
2019	Adult	+	+	+		+	+
2018	Adult	+	+			+	
2017	Adult	+				+	
2016	Juvenile	+			+	+	+
2015	Juvenile	+		+		+	+
	Juvenile	+	+	+		+	
2014	Adult	+	+	+		+	
	Adult	+	+	+		+	
	Adult	+	+	+		+	
	Adult	+	+	+		+	
2013	Adult	+	+	+		+	
	Juvenile	+	+	+		+	
	Adult	+	+	+		+	+
2011	Adult	+	+	+		+	
2010	Juvenile	+	+	+		+	
	Adult	+	+	+		+	
2009	Adult	+	+	+		+	
2007	Adult	+	+	+		+	+
2006	Adult	+	+			+	
	Adult						
2005	Adult	+	+			+	
2001	Adult	+	+	+		+	
2000	Juvenile						
	Adult	+	+			+	
1999	Adult	+	+			+	+
1996	Adult	+	+	+		+	+
1994	Adult						
1993	Adult	+	+	+		+	
1990	Adult						
1989	Adult						

**Fig. 2:**
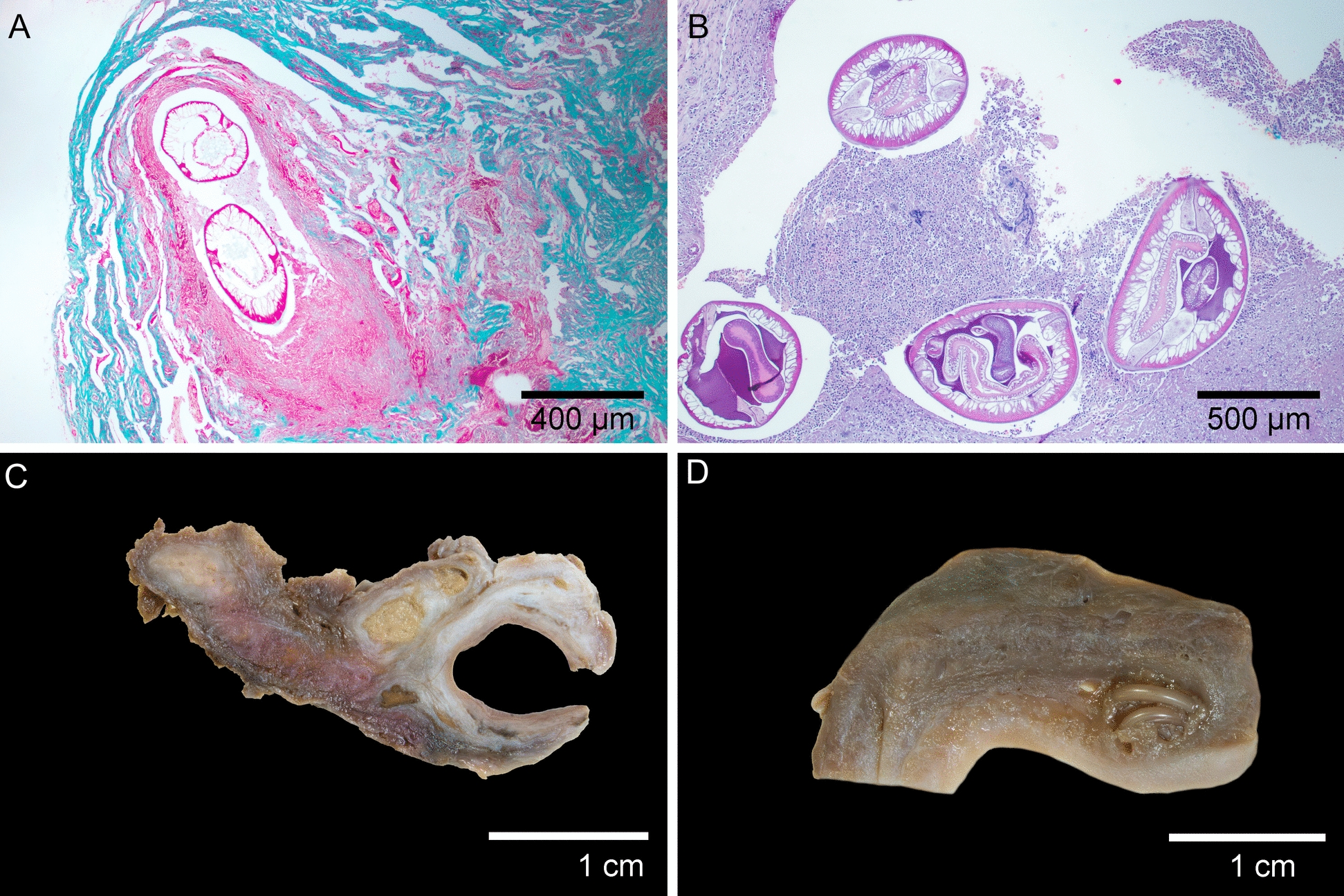
Histological and macroscopic lesions. **A** Migration through vascular walls causes severe fibrosis of the vascular wall (Masson’s trichrome stain). **B** Cross section of *Spirocerca lupi* adults in esophageal nodules with associated granulomatous inflammation, stained with hematoxylin and eosin. **C** Severe inflammation and proliferative granulation tissue in the aortic vascular wall associated with larval migration. Note the two migratory tracts filled with caseous material (formalin-fixed tissue). **D** Macroscopic view of an esophageal nodule containing *Spirocerca lupi* adults

**Fig. 3:**
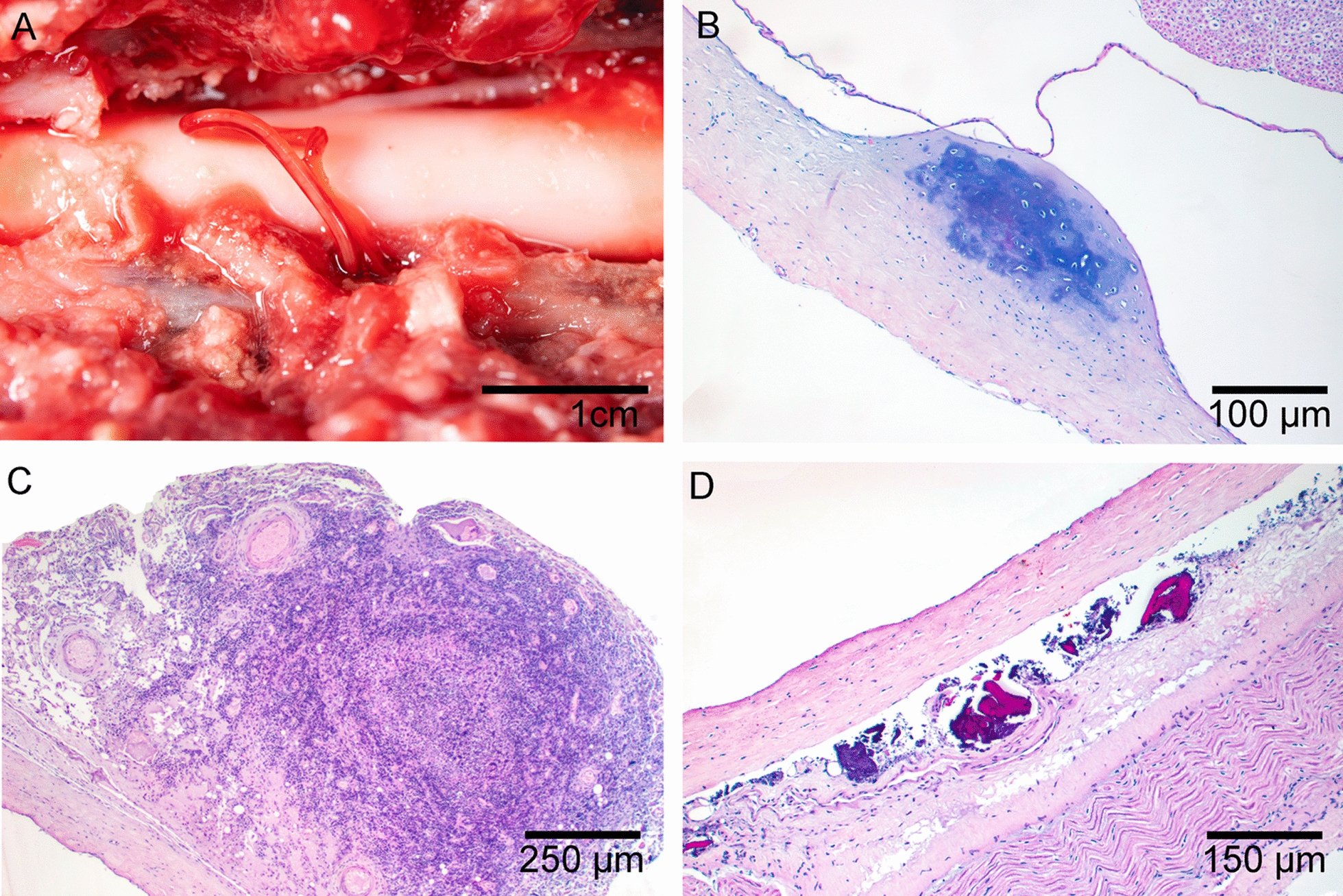
Vertebral lesions due to aberrant migration. **A** *Spirocerca* adult within the vertebral foramen adjacent to the spinal cord, observed after removal of the dorsal vertebral wall. **B** Focal cartilaginous metaplasia of the dura mater. **C** Granuloma formation associated with parasite migration. **D** Granulomatous infiltrate within the dura mater membrane

Histopathological examination revealed consistent lesion patterns across anatomical locations. In the aortic aneurysms, there was marked thickening of the vascular wall due to the proliferation of collagen fibers in the tunica intima and tunica media (Fig. 2A). Numerous macrophages were observed both perivascularly and intramurally. Lesions associated with larval migration were severe. When larvae were present, numerous epithelioid macrophages, eosinophils, and scattered lymphocytes surrounded the nematode larvae. In the absence of visible larvae, lesions typically displayed multifocal liquefactive necrosis (migration tracks) with abundant macrophages, degenerated neutrophils, and eosinophilic cellular debris. Granulomatous nodules on the vascular wall measured up to 1.5 cm in diameter (Fig. 2C). Esophageal nodules (Fig. 2D) were located beneath the epithelial layer of the mucosa and exhibited severe granulomatous and eosinophilic infiltration caused by the presence of adults. These nodules were encapsulated by fibrous stroma composed of disorganized collagen fibers, with small necrotic areas and scattered neutrophils. Each nodule featured a fistula connecting to the esophageal lumen. A notable finding in 16 of the 33 affected coyotes was interstitial pneumonia, characterized by diffuse alveolar damage, alveolar infiltration by neutrophils and macrophages, and type II pneumocyte hyperplasia. In one individual, aberrant larval migration into the spinal canal (Fig. 3A) resulted in chondroid metaplasia (Fig. 3B), localized neutrophilic inflammation of the dura mater (Fig. 3C), and mineralization. (Fig. 3D).

**Fig. 4:**
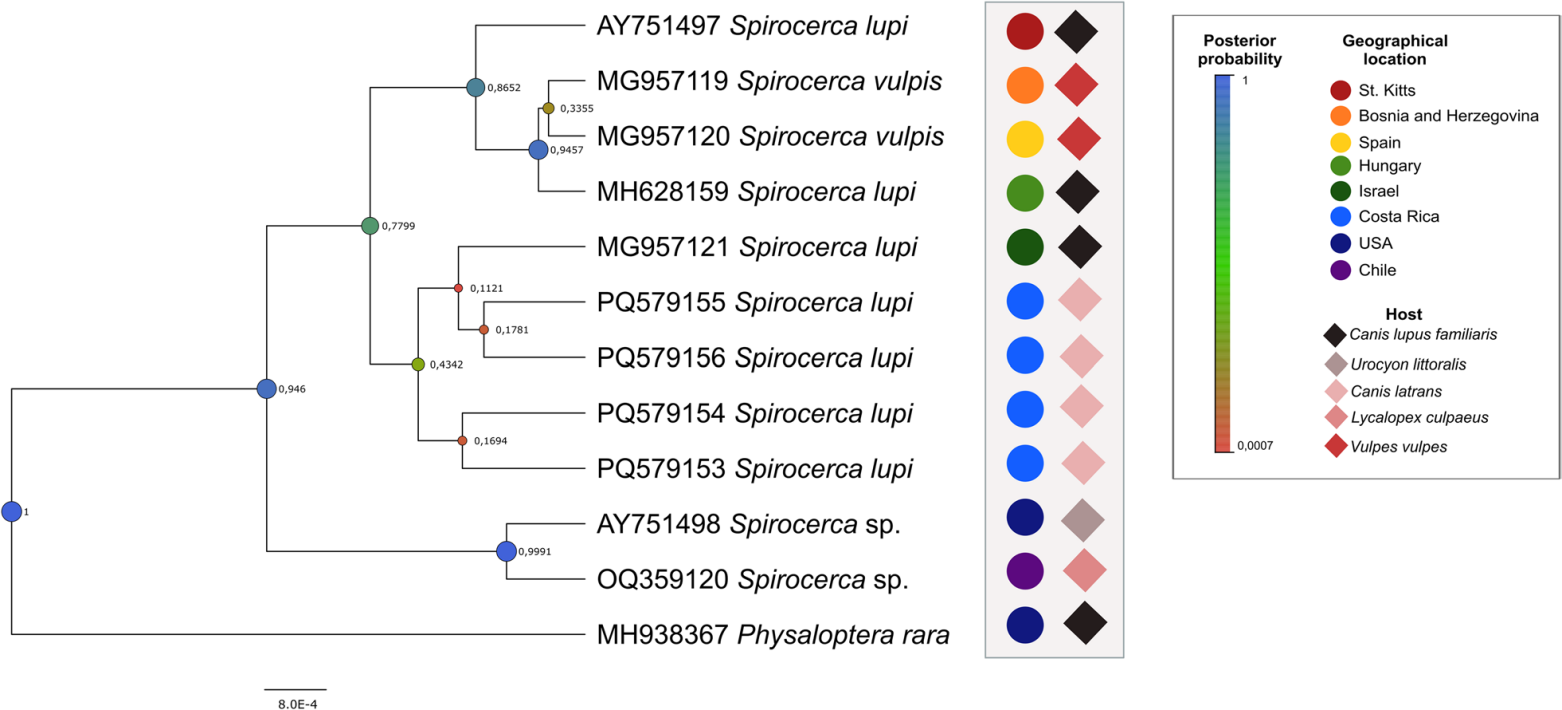
Bayesian inference phylogenetic tree of the *18S* gene of *Spirocerca lupi*. Posterior probability (PP) values are indicated next to each node. Node circle size and color are proportional to the PP

Using two statistical tests—the chi-squared test (Supplementary Fig. S1) to identify significant associations and Cramér’s V test (Supplementary Fig. S2) to measure their strength—we found statistically significant relationships between all analyzed pathological variables and *S. lupi* diagnostics. Cramér’s V strongly associated (> 0.75) granulomas with nematodes, suggesting that the finding of a nematode is almost always accompanied by a granuloma or vice versa. In addition, granulomas and aortic aneurysms are also strongly associated. The diagnostics of *S. lupi* are linked to granulomas/esophageal nodules and nematode findings, while other vascular and oncogenic developments are less strongly associated with *S. lupi* diagnostics (Fig. 4)

### Molecular analyses

Molecular identification based on *18S* rRNA of intralesional *Spirocerca* specimens was successful in all four recovered specimens, including the intravertebral individual. The sequences resulted were identical, and the consensus, with a length of 1665 bp, was submitted to GenBank under accession no. PV460829. This matched with 100% coverage and 99.82% identity to a *S. lupi* collected from a domestic dog from Saint Kitts (GenBank: AY751497). Phylogenetic analyses included all the *S. lupi* sequences available in GenBank for this gene. The *18S* sequences derived from the coyotes from Costa Rica were placed in a cluster with other sequences obtained from domestic dogs from different geographical regions and were closely related to *Spirocerca*.

**Fig. 5:**
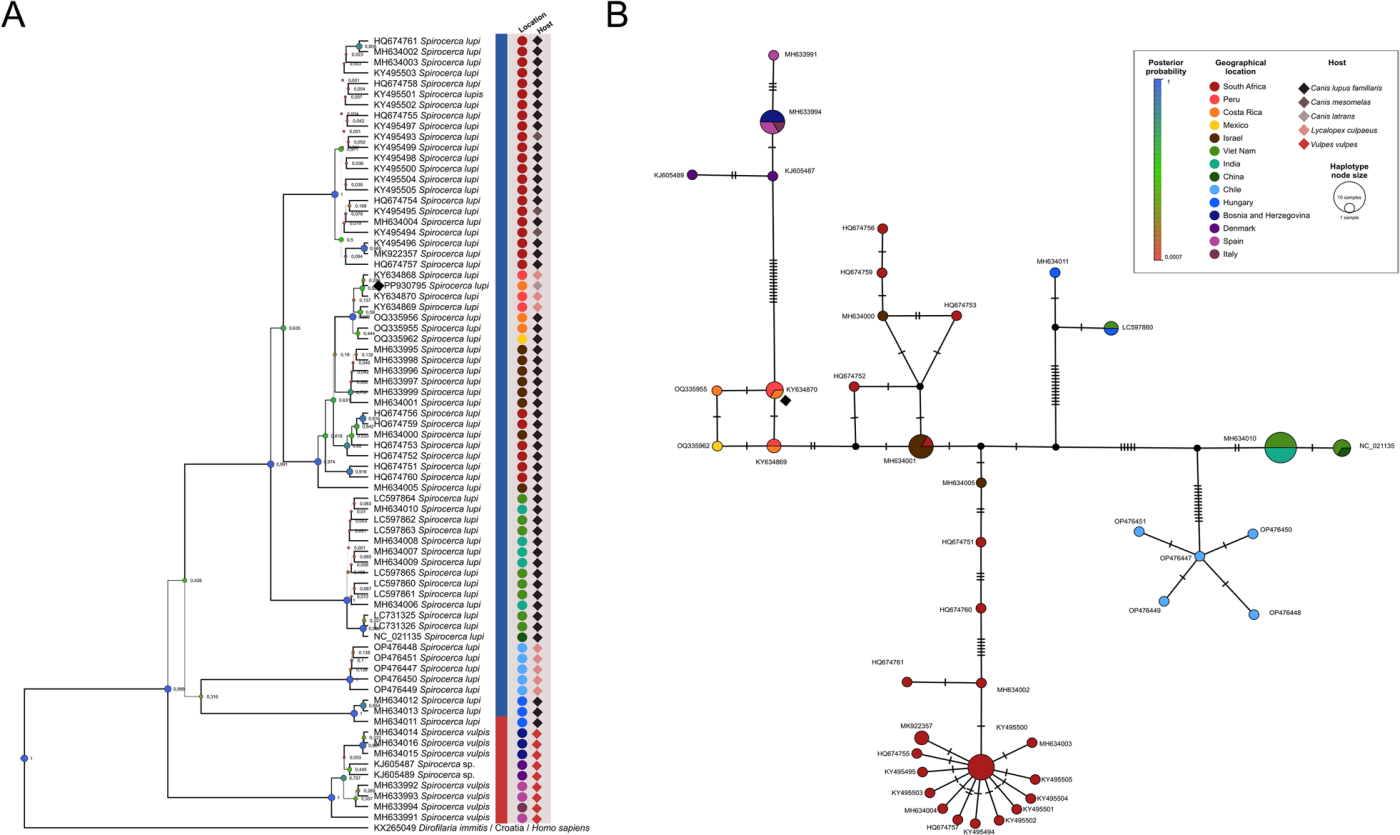
Bayesian inference phylogenetic tree and haplotype network of a *cox1* gene fragment of *Spirocerca lupi*. **A** *Dirofilaria immitis* was used as an outgroup and *Spirocerca vulpis* as a sister clade. Posterior probability values are next to each node, and node circle size and color are proportional to these values. The sequence derived from this study is indicated with a black diamond. **B** Templeton Crandall Sing haplotype network with 95% connection limit. Each haplotype is represented as a colored circle, hypothetical haplotypes as black circles, and mutations between haplotypes as hatch marks

Only one *cox1* sequence could be amplified from the worms, which was identical to *S. lupi* from Peru obtained from the Andean fox *Lycalopex culpaeus* (accession no. KY634870). The sequence derived from the coyote of Costa Rica was deposited in GenBank with accession no. PP930765. Phylogenetic analysis (Fig. 5a) placed the sequence from the coyote of Costa Rica in a clade with other sequences obtained from the Andean foxes from Peru and domestic dogs from Costa Rica and Mexico. Moreover, this clade was within genotype 1, which included *S. lupi* sequences derived from specimens of Israel, South Africa, India, Vietnam, and China. The haplotype network (Fig. 5b) replicated the findings of the phylogenetic tree, where the Costa Rican worm collected from the coyote was in the same haplotype as other sequences from the Andean foxes from Peru and next to other *S. lupi* worms from domestic dogs from Costa Rica and Mexico. This cluster from the Americas was next to a cluster with sequences from South Africa and Israel, rather than to other geographical locations.

In total, 13 (*n* = 13/137) fecal samples were positive for *S. lupi* DNA. Overall, 38% (5/13) of the positive samples were collected at the Santa Rosa National Park, whereas the other ones were obtained in the Central Conservation Area. All samples included in this study were confirmed as *Canis latrans* according to RFLP and 12S metabarcoding results (Supplementary Materials, Supplementary Table S1).

## Discussion

*Spirocerca*-associated lesions were a common necropsy finding in coyotes submitted to the PD-EMV in Costa Rica. The high frequency of these lesions (84%) is notably higher than that reported in domestic dogs and other wild carnivores. Reported *S. lupi* prevalence varies among studies; for example, rural dogs in Alabama, USA, showed a prevalence of 47% [36], while dogs in the Czech Republic [37] had a prevalence of only 0.2%. In Spain, *S. vulpis* was reported in 6% of examined foxes [38].

Lesions caused by *S. lupi* in wild carnivores are less consistent than those seen in domestic dogs, which commonly develop aortic aneurysms, esophageal granulomas, and soft tissue sarcomas. In contrast, wild carnivores tend to present primarily aortic aneurysms, while esophageal granulomas and sarcomas are less frequently observed. For example, in one study, only 7% of coyotes exhibited esophageal lesions, but 83% had aortic lesions [12].

The consistently high frequency of aortic lesions is noteworthy. One possible explanation for the variation in lesion patterns is an enhanced inflammatory response to larval migration in wild carnivores, which may impede larval progression toward the esophagus. Coyotes are New World canids, while *Spirocerca* has been demonstrated to have originated in Europe [13]. Considering this, *Spirocerca* might not have co-evolved with coyotes, potentially leading to greater resistance and lower tolerance to this parasite. This can be macroscopically observed as a severe aortic reaction (Fig. 2C). Nonetheless, this hypothesis needs to be supported by immunologically focused studies. The discrepancy between the prevalence of aortic lesions and the development of sarcomas is likely due to the timing of disease progression, since *Spirocerca* infection has a long timeframe. Aortic lesions typically manifest in the early stages of infection, whereas sarcoma formation occurs months or even years later. In one study from Mexico, only 3% of the coyotes analyzed presented esophageal nodules, and the authors considered that coyotes might be dead-end hosts, because of the low frequency of esophageal nodules in this species [39]. Nonetheless, based on our necropsy findings and fecal survey, coyotes are most likely a suboptimal host for *S. lupi*, because esophageal nodules and positive fecal samples are infrequent for this parasite. The present study presents the first report of spinal cord invasion by *S. lupi* larvae in coyotes, evidenced by larval invasion of the medullary cavity in one individual euthanized owing to loss of proprioception in the pelvic limbs. This aberrant pattern has been described previously in dogs [40]. Although this migration can trigger a severe inflammatory response in the host, surgical removal of nematodes in the extramedullary space has been reported as curative [41].

The chi-squared test and Cramér’s V test reinforce the idea that chronic inflammatory responses triggered by parasitic infections may contribute to oncogenesis, and in turn, that *S. lupi* in coyotes is being more commonly diagnosed in the chronic stage. This is also supported by the age variable, which was found to be statistically significantly associated with tumors, i.e., chronically infected animals [42]. Histopathology can offer a presumptive diagnosis with a high degree of certainty in the identification of intralesional parasites. The identification of *S. lupi* in histological slides relies on distinctive morphological traits, such as a unicellular, columnar epithelium-lined intestine and a pseudocoelom filled with eosinophilic material [43] (Fig. 2). However, these features alone might be suboptimal for definitive identification, as other species, such as *S. vulpis*, share similar morphology under light microscopy [44]. Nonetheless, this species has not been reported in the Americas. Therefore, molecular characterization is recommended for the accurate identification of *Spirocerca* spp. It is worth noting that the removal of *S. lupi* adults from lesions can be challenging. Depending on the larval stage, visualization with any magnification is not possible owing to the small size of the sub-adult nematodes. In addition, the removal of these can damage the tissue, making the histopathological report suboptimal. Therefore, this procedure is often avoided at necropsy, and the identification of the parasite relies only on the histopathological description.

Diagnosis of *S. lupi* in domestic dogs can be achieved through a combination of the animal’s clinical history and the identification of esophageal nodules using various imaging techniques, including endoscopy, ultrasonography, and computed tomography [5]. However, coprological methods remain cost-effective tools for diagnosing spirocercosis [2]. These methods rely on the morphological identification of eggs in fecal samples through direct examination or fecal flotation using a sugar solution [45]; however, molecular detection of *S. lupi* *18S *and *ITS1* loci in fecal samples is more sensitive than fecal flotation for identifying infected dogs [34]. Usually, molecular findings should be confirmed by morphological methods, i.e., observation of parasitic stages in clinical samples. Nevertheless, the high sensitivity demonstrated in previous studies, along with the confirmed sequences of *S. lupi* DNA, supports the validity of our current findings in coyotes.

Phylogenetic analysis of the recovered adults indicates an active distribution of the parasite among wild and domestic canid populations from the Americas. Phylogenetic analysis of the *cox1* gene fragment revealed a close similarity of the sequences derived from coyotes and domestic dogs from Costa Rica with other specimens collected from Andean foxes from Peru and domestic dogs from Mexico, consistent with previous findings [13]. This “American” cluster was closely related to other genotype 1 sequences from Israel, South Africa, India, China, and Vietnam, as analyzed before [24]. In one phylogeographic study, it was suggested that *S. lupi* may have been introduced to South America from Israel and subsequently spread to Central and North America [13]. The latter study had the limitation of no sequence availability from other geographical locations. On the other side, *18S* gene analysis included a few different sequences owing to its low representation in GenBank and presented very low PP values in the phylogenetic tree, which is explained by the high degree of conservation of this gene among nematodes [46]. The *18S* analysis ultimately gave very limited information about the phylogenetic positioning of *S. lupi* sequences obtained herein, as opposed to the *cox1* study. The current evidence does not support the hypothesis that coyotes were responsible for introducing *Spirocerca* into South America. On the contrary, coyotes have been expanding from North America into Central and South America since the early 1900s [15]. Now established in these regions, coyotes likely play a significant role in the parasite’s transmission dynamics. The domestic dogs, as well as paratenic or intermediate host trade, might have been responsible for such introduction to the Americas. Further research is needed to understand better the ecological interactions between *S. lupi* and coyote populations.

The elevated infection rate among coyotes suggests that *S. lupi* is widespread within wild populations. Coyotes may be more susceptible to *S. lupi* infection owing to their increased likelihood of consuming infected paratenic hosts compared with domestic dogs [47, 48]. Their diet predominantly consists of small mammals, lagomorphs, and insects, particularly beetles (Coleoptera). Various species of small mammals and rabbits [2, 49] have been identified as paratenic hosts of *S. lupi*, with transmission typically occurring through the ingestion of these hosts rather than direct consumption of beetles.

Currently, no clear evidence defines the direction of parasite transmission between hosts, leaving it uncertain whether *S. lupi* is primarily transmitted from domestic dogs to coyotes, vice versa, or whether they both function as a maintenance community for this parasite [50]. The high prevalence observed in necropsied animals suggests that spirocercosis may have a detrimental impact on coyote fitness and survival in the wild. However, the extent to which this parasite affects the Costa Rican coyote population remains unclear. Our detection of *S. lupi* in 4.6% of coyote fecal samples suggests that the parasite is not widespread among the population. Nevertheless, its consistent diagnosis in coyotes over several years strongly indicates that the parasite is well established in wild carnivores. Continued surveillance of coyotes, gray foxes, and other potential hosts in Costa Rica is essential to understanding the ecological impact of *S. lupi* and its potential for cross-species transmission.

## Conclusions

Determining the prevalence of *S. lupi* infection in wild populations is crucial, along with assessing the age of infection, as individuals appear to harbor the parasite for extended periods, potentially leading to chronic complications and even mortality. In sum, chronic inflammatory responses triggered by parasitic infections contribute to oncogenesis, particularly in esophageal or fibrosarcomatous developments, and point to a cardiovascular risk in chronic nematode infections that might cause mortality at chronic stages of the disease. In addition, coyotes may serve as a reservoir for the disease, posing a transmission risk to other carnivores, including domestic species.

## Supplementary Information

Supplementary Material 1.

## Data Availability

Data supporting the main conclusions of this study are included in the manuscript.
