# Supplementary material for: Retrospective study of spirocercosis in coyotes (Canis latrans) in Costa Rica: epidemiology, pathological findings, and molecular characterization of Spirocerca lupi
Source: Parasit Vectors. 2025 Oct 27;18:431. doi: 10.1186/s13071-025-07030-4 (PMC12560332; doi:10.1186/s13071-025-07030-4)
Supplement: Supplementary file 1 — Supplementary Material 1. [file 13071_2025_7030_MOESM1_ESM.docx]

**Supplementary Information**

**Additional file 1: Table S1.** Positive fecal samples from Coyotes. P.N.= Parque Nacional (National Park) P.N.V.= Parque Nacional Volcán (Volcano National Park), P.N.V.I.= Parque Nacional Volcán Irazu (Irazu Volcano National Park).

| **Sample** | ***PCR S. lupi*** | **Sampling date** | **Collection Site** |
| --- | --- | --- | --- |
| CY1 | **POSITIVE** | 7/4/21 | Cerro Muerte |
| CY2 | NEGATIVE | 12/5/21 | Guanacaste(P.N.Guanacaste) |
| CY3 | NEGATIVE | 6/6/21 | P.N.V.I Prusia |
| CY4 | NEGATIVE | 6/6/21 | P.N.V.I Prusia |
| CY5 | **POSITIVE** | 6/6/21 | P.N.V.I Prusia |
| CY6 | NEGATIVE | 6/6/21 | P.N.V.I Prusia |
| CY7 | NEGATIVE | 6/6/21 | P.N.V.I Prusia |
| CY8 | **POSITIVE** | 6/6/21 | P.N.V.I Prusia |
| CY11 | **POSITIVE** | 6/6/21 | P.N.V.I Prusia |
| CY15 | NEGATIVE | 6/6/21 | P.N.V.I Prusia |
| CY17 | NEGATIVE | 6/6/21 | P.N.V.I Prusia |
| CY18 | NEGATIVE | 6/6/21 | P.N.V.I Prusia |
| CY19 | NEGATIVE | 6/6/21 | P.N.V.I Prusia |
| CY21 | NEGATIVE | 6/6/21 | P.N.V.I Prusia |
| CY22 | NEGATIVE | 6/6/21 | P.N.V.I Prusia |
| CY48 | NEGATIVE | 6/6/21 | P.N.V.I Prusia |
| CY69 | NEGATIVE | 16/7/21 | P.N.V.Turrialba |
| CY70 | NEGATIVE | 16/7/21 | P.N.V.Turrialba |
| CY71 | NEGATIVE | 16/7/21 | P.N.V.Turrialba |
| CY74 | NEGATIVE | 16/7/21 | P.N.V.Turrialba |
| CY76 | NEGATIVE | 16/7/21 | P.N.V.Turrialba |
| CY79 | **POSITIVE** | 16/7/21 | P.N.V.Turrialba |
| CY80 | NEGATIVE | 16/7/21 | P.N.V.Turrialba |
| CY81 | **POSITIVE** | 16/7/21 | P.N.V.Turrialba |
| CY82 | NEGATIVE | 16/7/21 | P.N.V.Turrialba |
| CY83 | NEGATIVE | 16/7/21 | P.N.V.Turrialba |
| CY87 | NEGATIVE | 16/7/21 | P.N.Tapantí-Macizo Muerte |
| CY88 | NEGATIVE | 16/7/21 | P.N.Tapantí-Macizo Muerte |
| CY89 | NEGATIVE | 16/7/21 | P.N.Tapantí-Macizo Muerte |
| CY91 | **POSITIVE** | 21/7/21 | P.N.Santa Rosa |
| CY92 | **POSITIVE** | 21/7/21 | P.N.Santa Rosa |
| CY93 | NEGATIVE | 21/7/21 | P.N.Santa Rosa |
| CY94 | **POSITIVE** | 21/7/21 | P.N.Santa Rosa |
| CY95 | NEGATIVE | 21/7/21 | P.N.Santa Rosa |
| CY96 | **POSITIVE** | 21/7/21 | P.N.Santa Rosa |
| CY97 | NEGATIVE | 21/7/21 | P.N.Santa Rosa |
| CY98 | NEGATIVE | 21/7/21 | P.N.Santa Rosa |
| CY99 | **POSITIVE** | 21/7/21 | P.N.Santa Rosa |
| CY100 | NEGATIVE | 16/7/21 | P.N.Tapantí-Macizo Muerte |
| CY101 | NEGATIVE | 28/7/21 | P.N.VI. Irazú Crateres |
| CY102 | NEGATIVE | 28/7/21 | P.N.VI. Irazú Crateres |
| CY103 | NEGATIVE | 28/7/21 | P.N.VI. Irazú Crateres |
| CY108 | NEGATIVE | 10/9/21 | P.N.V.I Prusia |
| CY109 | **POSITIVE** | 10/9/21 | P.N.V.I Prusia |
| CT110 | NEGATIVE | 10/9/21 | P.N.V.I Prusia |
| CY111 | **NEGATIVE** | 10/9/21 | P.N.V.I Prusia |
| CY112 | NEGATIVE | 10/9/21 | P.N.V.I Prusia |
| CY113 | NEGATIVE | 10/9/21 | P.N.V.I Prusia |
| CY114 | NEGATIVE | 10/9/21 | P.N.V.I Prusia |
| CY115 | NEGATIVE | 10/9/21 | P.N.V.I Prusia |
| CY116 | NEGATIVE | 10/9/21 | P.N.V.I Prusia |
| CY118 | NEGATIVE | 10/9/21 | P.N.V.I Prusia |
| CY119 | NEGATIVE | 10/9/21 | P.N.VI. Irazú Crateres |
| CY120 | NEGATIVE | 10/9/21 | P.N.VI. Irazú Crateres |
| CY121 | NEGATIVE | 10/9/21 | P.N.VI. Irazú Crateres |
| CY122 | NEGATIVE | 10/9/21 | P.N.VI. Irazú Crateres |
| CY123 | NEGATIVE | 10/9/21 | P.N.VI. Irazú Crateres |
| CY124 | NEGATIVE | 10/9/21 | P.N.VI. Irazú Crateres |
| CY125 | NEGATIVE | 10/9/21 | P.N.VI. Irazú Crateres |
| CY126 | NEGATIVE | 10/9/21 | P.N.VI. Irazú Crateres |
| CY127 | NEGATIVE | 10/9/21 | P.N.VI. Irazú Crateres |
| CY128 | NEGATIVE | 10/9/21 | P.N.VI. Irazú Crateres |
| CY129 | NEGATIVE | 10/9/21 | P.N.VI. Irazú Crateres |
| CY130 | NEGATIVE | 10/9/21 | P.N.VI. Irazú Crateres |
| CY131 | **POSITIVE** | 10/9/21 | P.N.VI. Irazú Crateres |
| CY132 | NEGATIVE | 10/9/21 | P.N.VI. Irazú Crateres |
| CY133 | NEGATIVE | 10/9/21 | P.N.VI. Irazú Crateres |
| CY134 | NEGATIVE | 10/9/21 | P.N.VI. Irazú Crateres |
| CY135 | NEGATIVE | 10/9/21 | P.N.VI. Irazú Crateres |
| CY136 | NEGATIVE | 10/9/21 | P.N.VI. Irazú Crateres |
| CY137 | NEGATIVE | 10/9/21 | P.N.VI. Irazú Crateres |
| CY140 | NEGATIVE | 10/9/21 | P.N.V.I Prusia |
| CY141 | NEGATIVE | 10/9/21 | P.N.V.I Prusia |
| CY142 | NEGATIVE | 10/9/21 | P.N.V.I Prusia |
| CY143 | NEGATIVE | 10/9/21 | P.N.V.I Prusia |
| CY144 | NEGATIVE | 24/9/21 | La Estefana |
| CY145 | NEGATIVE | 24/9/21 | La Estefana |
| CY146 | NEGATIVE | 24/9/21 | La Estefana |
| CY147 | NEGATIVE | 24/9/21 | La Estefana |
| CY148 | NEGATIVE | 24/9/21 | La Estefana |
| CY149 | NEGATIVE | 24/9/21 | La Estefana |
| CY150 | NEGATIVE | 24/9/21 | La Estefana |
| CY151 | NEGATIVE | 24/9/21 | La Estefana |
| CY152 | NEGATIVE | 24/9/21 | La Estefana |
| CY153 | NEGATIVE | 24/9/21 | La Estefana |
| CY154 | NEGATIVE | 24/9/21 | La Estefana |
| CY155 | NEGATIVE | 24/9/21 | La Estefana |
| CY156 | NEGATIVE | 24/9/21 | La Estefana |
| CY157 | NEGATIVE | 24/9/21 | La Estefana |
| CY158 | NEGATIVE | 24/9/21 | La Estefana |
| CY159 | NEGATIVE | 24/9/21 | La Estefana |
| CY160 | NEGATIVE | 24/9/21 | La Estefana |
| CY161 | NEGATIVE | 24/9/21 | La Estefana |
| CY162 | NEGATIVE | 24/9/21 | La Estefana |
| CY163 | NEGATIVE | 24/9/21 | La Estefana |
| CY164 | NEGATIVE | 24/9/21 | La Estefana |
| CY165 | NEGATIVE | 24/9/21 | La Estefana |
| CY166 | NEGATIVE | 24/9/21 | La Estefana |
| CY167 | NEGATIVE | 24/9/21 | La Estefana |
| CY169 | NEGATIVE | 24/9/21 | La Estefana |
| CY170 | NEGATIVE | 24/9/21 | La Estefana |
| CY171 | NEGATIVE | 24/9/21 | La Estefana |
| CY172 | NEGATIVE | 24/9/21 | La Estefana |
| CY174 | NEGATIVE | 15/10/21 | P.N.V.I |
| CY177 | NEGATIVE | 15/10/21 | P.N.V.I |
| CY178 | NEGATIVE | 15/10/21 | P.N.V.I |
| CY179 | NEGATIVE | 15/10/21 | P.N.V.I |
| CY181 | NEGATIVE | 15/10/21 | P.N.V.I |
| CY183 | NEGATIVE | 15/10/21 | P.N.V.I |
| CY184 | NEGATIVE | 15/10/21 | P.N.V.I |
| CY186 | NEGATIVE | 15/10/21 | P.N.V.I |
| CY187 | NEGATIVE | 15/10/21 | P.N.V.I |
| CY188 | NEGATIVE | 15/10/21 | P.N.V.I |
| CY189 | NEGATIVE | 15/10/21 | P.N.V.I |
| CY190 | NEGATIVE | 15/10/21 | P.N.V.I |
| CY192 | NEGATIVE | 15/10/21 | P.N.V.I |
| CY195 | NEGATIVE | 15/10/21 | P.N.V.I |
| CY196 | NEGATIVE | 15/10/21 | P.N.V.I |
| CY198 | NEGATIVE | 15/10/21 | P.N.V.I |
| CY200 | NEGATIVE | 15/10/21 | P.N.V.I |
| CY201 | NEGATIVE | 15/10/21 | P.N.V.I |
| CY202 | NEGATIVE | 15/10/21 | P.N.V.I |
| CY203 | NEGATIVE | 15/10/21 | P.N.V.I |
| CY204 | NEGATIVE | 15/10/21 | P.N.V.I |
| CY206 | NEGATIVE | 15/10/21 | P.N.V.I |
| CY207 | NEGATIVE | 15/10/21 | P.N.V.I |
| CY208 | NEGATIVE | 15/10/21 | P.N.V.I |
| CY209 | NEGATIVE | 15/10/21 | P.N.V.I |
| CY211 | NEGATIVE | 15/10/21 | P.N.V.I |
| CY212 | NEGATIVE | 15/10/21 | P.N.V.I |
| CY213 | NEGATIVE | 20/11/21 | P.N.Santa Rosa |
| CY214 | NEGATIVE | 20/11/21 | P.N.Santa Rosa |
| CY215 | NEGATIVE | 20/11/21 | P.N.Santa Rosa |
| CY216 | NEGATIVE | 20/11/21 | P.N.Santa Rosa |
| CY217 | NEGATIVE | 20/11/21 | P.N.Santa Rosa |
| CY218 | NEGATIVE | 20/11/21 | P.N.Santa Rosa |
| CY220 | NEGATIVE | 20/11/21 | P.N.Santa Rosa |

**Additional file 2: Figure S1.** Chi-Square Test.

**Additional file 3: Figure S2**. Cramer´s V Correlation Matrix.
